# Biophysical feedbacks mediate carbonate chemistry in coastal ecosystems across spatiotemporal gradients

**DOI:** 10.1038/s41598-017-18736-6

**Published:** 2018-01-15

**Authors:** Nyssa J. Silbiger, Cascade J. B. Sorte

**Affiliations:** 1 Department of Ecology and Evolutionary Biology, University of California, Irvine, 321 Steinhaus Hall, Irvine, CA 92697 USA; 2 Department of Biology, California State University, Northridge, 18111 Nordhoff Street, Northridge, CA 91330 USA

## Abstract

Ocean acidification (OA) projections are primarily based on open ocean environments, despite the ecological importance of coastal systems in which carbonate dynamics are fundamentally different. Using temperate tide pools as a natural laboratory, we quantified the relative contribution of community composition, ecosystem metabolism, and physical attributes to spatiotemporal variability in carbonate chemistry. We found that biological processes were the primary drivers of local pH conditions. Specifically, non-encrusting producer-dominated systems had the highest and most variable pH environments and the highest production rates, patterns that were consistent across sites spanning 11° of latitude and encompassing multiple gradients of natural variability. Furthermore, we demonstrated a biophysical feedback loop in which net community production increased pH, leading to higher net ecosystem calcification. Extreme spatiotemporal variability in pH is, thus, both impacting and driven by biological processes, indicating that shifts in community composition and ecosystem metabolism are poised to locally buffer or intensify the effects of OA.

## Introduction

Ocean acidification (OA) is a significant and increasing threat to marine ecosystems^1–3^. One-third of anthropogenic CO_2_ emissions have been absorbed by the ocean^4^, leading to a decrease in ocean pH of ~0.1 pH units since pre-industrial times^5–7^. To date, OA projections and experimental scenarios are based primarily on well-mixed, open ocean conditions^5,8–10^, but carbonate dynamics in coastal ecosystems are fundamentally different^9^. Understanding these dynamics is essential given that OA threatens many goods and services provided by coastal ecosystems^11^. Despite representing only a small fraction of the ocean (~7% is shallower than 200 m), coastal ecosystems contribute disproportionately to marine primary production (~25% of the ocean total) and fisheries catch (~90%)^12,13^. Coastal systems are also disproportionately impacted by humans as the majority of the world’s population lives within 100 km of coastlines^14^. Given the rising threat of OA to vital coastal resources, there is a fundamental need to understand the drivers of spatiotemporal variability in carbonate chemistry in coastal ecosystems.

Coastal ecosystems are embedded in a highly dynamic pH environment in which biological processes can strongly influence local pH conditions^9,15,16^. Within a single day, pH can range from 7.6 to 8.0 on a shallow coral reef^16^ and from 7.2 to 9.0 in a temperate intertidal zone^17,18^. These ranges are, respectively, 2 and 9 times greater than the predicted “end-of-the-century” change in global pH^5,8^. This level of natural variability likely reflects biological processes, which can both drive and respond to changes in ocean pH, thereby creating biophysical feedback loops. For example, OA enhances photosynthesis in many fleshy macroalgal species and seagrasses^19^, and photosynthesis and respiration have opposing effects on CO_2_ concentration, increasing and decreasing pH, respectively. OA also typically decreases calcification in organisms with CaCO_3_ shells or skeletons^20,21^. Furthermore, calcification and dissolution also drive pH through shifts in the concentration of dissolved inorganic carbon and total alkalinity in which calcification decreases pH while dissolution increases it^22^. Therefore, shifts in metabolic processes in response to OA could feed back to further change the local biogeochemical environment.

Both positive and negative feedbacks between carbonate chemistry and reef metabolism have been demonstrated on coral reefs^20,23–26^. For example, along the Florida Reef Tract, seasonal changes in net photosynthesis and net respiration led to seasonal fluctuations in aragonite saturation state (Ω_arag_) and, thus, pH^27^. The shifting Ω_arag_ drove changes in net calcification and dissolution and, in turn, the seasonal changes in calcification and dissolution offset changes in Ω_arag_ creating positive feedbacks in the spring/summer (higher net photosynthesis leading to higher net calcification) and negative feedbacks in the fall/winter (higher net respiration leading to lower net calcification, and, in some locations, even net dissolution)^28^. While biological feedbacks have been described on coral reefs, latitudinal patterns in community metabolism and biological feedbacks are less well known in temperate ecosystems.

Community composition is set to play an important role in driving positive or negative feedbacks in local pH conditions^29^, given species’ differing roles in ecosystem metabolism and impacts of shifting CO_2_ environments on benthic communities^3,28,30^. For example, autotrophs (producers) drive increases in pH during the day through photosynthesis and decreases at night, whereas heterotrophs (consumers) always decrease pH by respiring CO_2_. Biological feedbacks with pH are also likely to depend on the context of covarying physical (e.g., light, temperature, residence time) and chemical factors (e.g., dissolved oxygen) which can affect community interactions and metabolic rates^31,32^. As coastal habitats with high producer biomass are declining globally^9,33^, it is critical that we understand how shifts in community composition will impact local pH environments in the context of natural and highly variable coastal ecosystems.

**Figure 1:**
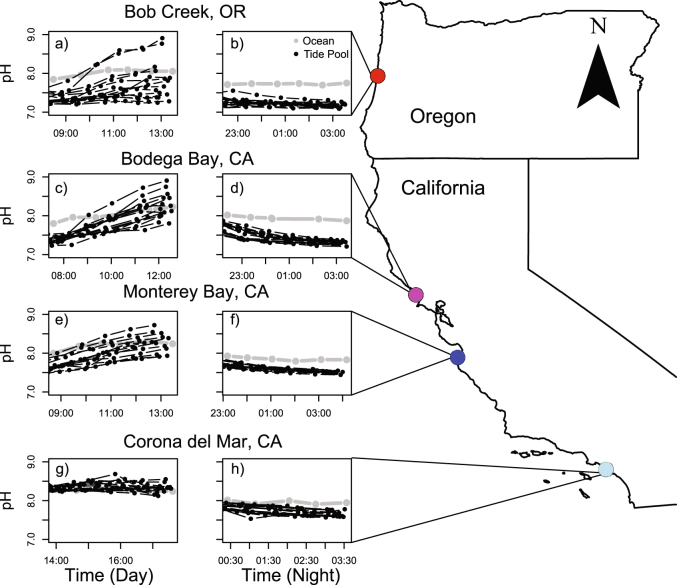
Seawater pH at sites on the U.S. West Coast. pH (total scale) over time during the (**a**,**c**,**e**,**g**) daytime and (**b**,**d**,**f**,**h**) nighttime sampling for (**a**,**b**) Bob Creek, OR, (**c**,**d**) Bodega Bay, CA, (**e**,**f**) Monterey Bay, CA, and (**g**,**h**) Corona del Mar, CA. Data are from tide pools (black) and the adjacent ocean (grey). The map was made with ArcGIS (ESRI. 2015. ArcGIS Desktop: Release 10.4.1. Redlands, CA: Environmental Systems Research Institute, www.arcgis.com).

The goals of this study were to evaluate the relationships and feedbacks between community composition, ecosystem metabolism, and local pH conditions to, ultimately, determine the potential for local buffering or intensification of OA. Natural *in situ* studies are essential for assessing context-dependency of biological processes^34^. Tide pools can be used as natural laboratories for addressing these goals because they are essentially closed systems during low tides; therefore, biogeochemical changes are primarily driven by quantifiable local processes. Within 57 tide pools spanning four sites from southern California to central Oregon, USA (Fig. 1), we characterized community composition and physical attributes, and we quantified ecosystem metabolism during daytime and nighttime sampling events. Specifically, we (i) tested the relative effect of producer dominance (the relative abundance of non-encrusting producers versus consumers), ecosystem metabolism, tide pool physical attributes, temperature, and light availability on local pH conditions (mean and variability), and (ii) determined the importance of biological feedbacks between pH and key metabolic rates, particularly net community production (NCP = gross primary production − respiration) and net ecosystem calcification (NEC = gross calcification − gross CaCO_3_ dissolution). These findings underscore the importance of understanding biophysical feedbacks in coastal ecosystems in order to predict whether biological processes will locally buffer or amplify the impacts of ocean acidification.

## Results

### Tide Pool Characterization

**Figure 2:**
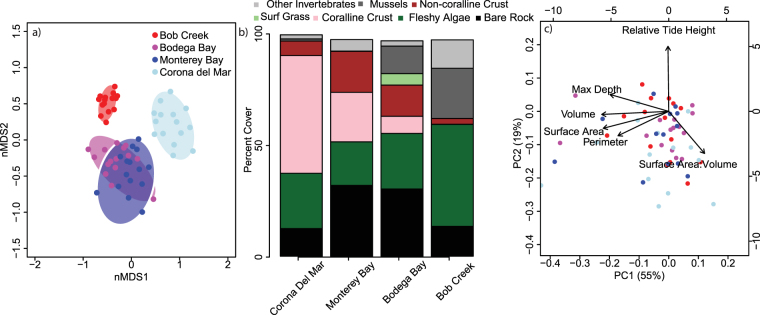
Community composition and physical attributes of tide pool systems. (**a**) NMDS plot of community composition across tide pools grouped by site (2D stress = 0.19). (**b**) Average relative abundance (percent cover) of functional group across n = 13–15 tide pools per site. Colors represent functional groups with invertebrates in grey (dark grey is the percent of invertebrates that are mussels), non-coralline crust in red, surf grass in light green, coralline algae in pink, fleshy algae in dark green, and bare rock in black. (**c**) PCA of tide pool physical attributes. Values are PC scores for individual tide pools colored by site, and arrows indicate loadings of each variable. PC axes 1 and 2 explain 55% and 19% of the variance.

Tide pools were characterized in terms of both their biological communities and their physical characteristics. The tide pool communities varied substantially both within sites and across the latitudinal gradient (Fig. 2, Table S1). The 57 tide pool communities were most similar within sites, with the two mid-coast sites (Bodega Bay, CA and Monterey Bay, CA) having the greatest overlap in species composition (Fig. 2a). Across the latitudinal gradient, invertebrates (mostly the mussel *Mytilus californianus*) and fleshy algae increased, while crustose coralline cover decreased, with increasing latitude (Fig. 2b). Overall, the biomass of non-encrusting producers and consumers both increased from south to north. Our PCA analysis of physical tide pool attributes indicated that PC1 was driven primarily by pool size (perimeter, max depth, surface area, volume, surface area-to-volume ratio) and explained 55% of the variation in the data, while PC2 was driven by differences in pool location with respect to the ocean (relative tide height) and explained 19% of the variation (Fig. 2c). These physical attributes varied within sites, but there were no differences in physical characteristics when they were compared across the four sites (Fig. 2c, Table S2).

### pH Variability Across Spatiotemporal Gradients

There was marked pH variability both within and across tide pools (Fig. 1) as evident from daytime and nighttime low tide water sampling events. Diel pH variability during low tide ranged from 0.5 to 1.1 at Corona del Mar, 0.4 to 1.2 at Monterey Bay, 0.5 to 1.7 at Bodega Bay, and 0.1 to 1.8 units at Bob Creek (Table S2). Mean pH also varied considerably between daytime (7.20–8.90) and nighttime (7.09–7.78) sampling events across all 57 tide pools. Additionally, there was substantial spatial variability in the adjacent ocean pH samples. Average ocean pH decreased with increasing latitude, from 8.12 at Corona del Mar to 7.87 at Bob Creek (Table S2). However, tide pool pH was largely de-coupled from adjacent ocean pH during low tide. The site with the lowest oceanic pH (Bob Creek) was also the site with the highest pH recorded across all 57 tide pools (8.9 pH units; Fig. 1). Values for the remaining carbonate parameters are available in supplemental Table S2.

### Biological versus Physical Drivers of pH

**Figure 3:**
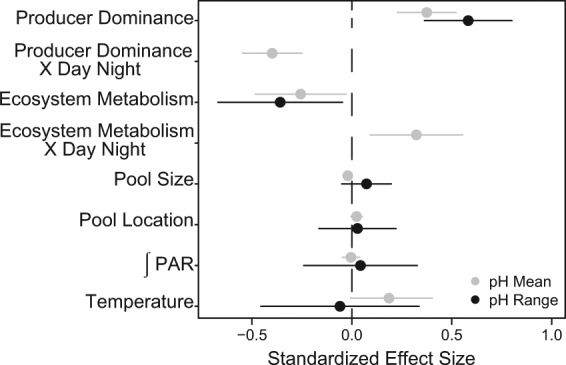
Relative effect of biological and physical parameters on mean and range in tide pool pH. Standardized effect sizes for the pH mean (Table S4; grey) and the pH range (Table S3; black) models. Predictor variables are producer dominance (% cover of non-encrusting producers – % cover of consumers), ecosystem metabolism (TA/DIC slopes), pool size (PC1; Fig. 3), pool location (PC2; Fig. 3), log-transformed integrated PAR, and temperature (mean and range for the mean and range pH models, respectively). The pH mean model also included an interaction term for time of day (day or night) with producer dominance and ecosystem metabolism. Values are effect sizes ± 95% CI. All predictors and response variables were standardized, and values with 95% CIs that do not cross zero are considered statistically significant.

**Figure 4:**
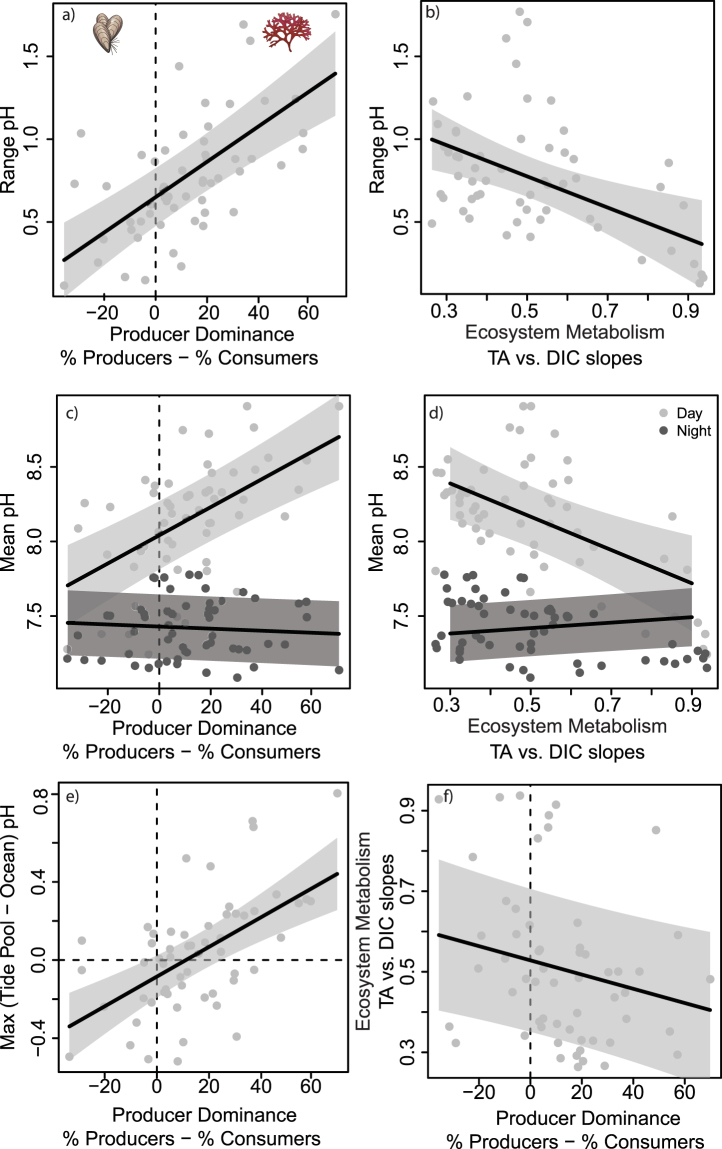
Relationships between producer dominance and ecosystem metabolism with pH in **(a,b)** range and **(c,d)** mean pH models. pH was influenced by (**a**,**c**) producer dominance (% cover of non-encrusting producers – % cover of consumers) and (**b**,**d**) ecosystem metabolism (TA/DIC slopes). Mean pH values (**c**,**d**) are shown for day (light grey) and night (dark grey) sampling. Panel (e) shows the relationship between producer dominance and pH divergence (the max difference in pH between the tide pool and the adjacent ocean sample). Panel (f) shows the relationship between ecosystem metabolism and producer dominance. ANOVA and summary results for all models are in Tables S3–S6. Values are from n = 57 tide pools, with best-fit lines and shaded 95% CIs. Symbols are Courtesy of the Integration and Application Network, University of Maryland Center for Environmental Science (ian.umces.edu/symbols/).

We tested the relative effect of non-encrusting producer dominance (fleshy macroalgae + surf grass − invertebrates; hereafter referred to as “producer dominance”), ecosystem metabolism, light availability, temperature, and tide pool physical attributes on pH mean and range across the latitudinal gradient. The biological parameters (producer dominance and ecosystem metabolism) were consistently the primary drivers of local pH conditions (Fig. 3). Specifically, non-encrusting producer-dominated pools had the highest and most variable pH environments (*P* < 0.001, Figs 3 and 4a,c, Tables S3, S4), whereas consumer-dominated pools had lower pH values, with minimal differences in pH between day and night across the latitudinal gradient (*P* < 0.001, Fig. 4c). Further, producer dominance caused a divergence between tide pool and ocean pH (*P* < 0.001, Fig. 4e). Specifically, for every 10% increase in producer dominance, there was a 0.08 unit increase in pH relative to the ocean (Fig. 4e, Table S5). pH conditions were also driven by ecosystem metabolism (Fig. 3, Tables S3, S4), defined here as the relationship between total alkalinity (TA) and dissolved inorganic carbon (DIC). As we expected, tide pools with more non-encrusting producers had lower TA/DIC slopes due to high rates of NCP (*P* = 0.009, Fig. 4f, Table S6), and lower TA/DIC slopes were associated with more variable pH environments (*P* = 0.03, Fig. 4b). The most productive pools also had increased mean pH relative to other pools during the day but not at night (Day/Night interaction *P* = 0.01, Figs 3, 4d). None of the physical parameters significantly affected pH range (Fig. 3, Table S3). However, there was a slightly positive effect of temperature on mean pH (*P* = 0.05, Fig. 3, Table S4) and a significant relationship between mean pH and pool size (*P* = 0.01, Fig. 3, Table S4). Even so, the effect of pool size was negligible compared to producer dominance and ecosystem metabolism, whose impacts on pH were, respectively, 17.5 and 13.5 times higher.

**Figure 5:**
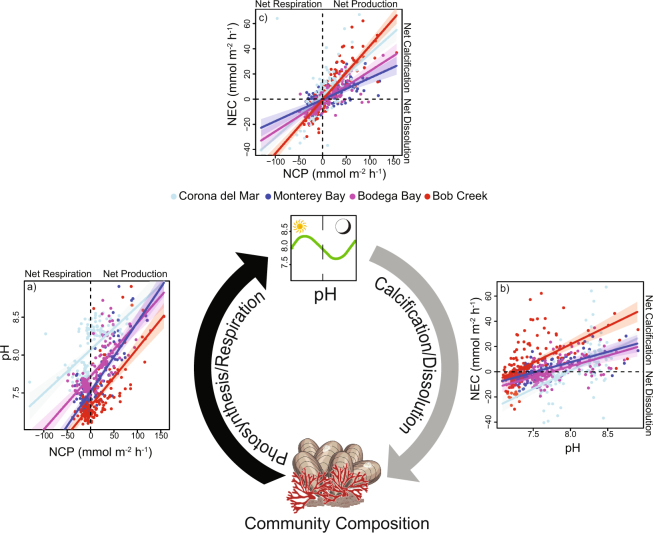
Feedback loop between pH and ecosystem functioning in coastal ecosystems. Community composition and ecosystem metabolism influence pH via photosynthesis and respiration rates (black arrow). NCP drives pH, in turn influencing calcification and dissolution (grey arrow). Our results show that (**a**) net community production (NCP) has a positive effect on pH (Table S7), (**b**) pH has a positive effect on net ecosystem calcification (NEC) (Table S8), and (**c**) NCP has a positive effect on NEC (Table S9). Values are for individual tide pools, colored by site. Symbols are Courtesy of the Integration and Application Network, University of Maryland Center for Environmental Science (ian.umces.edu/symbols/).

We uncovered strong biophysical feedbacks at all four study sites, with NCP and NEC both driving and responding to changes in pH. Specifically, CO_2_ fixation (NCP) increased pH (*P* < 0.0001, Fig. 5a and Table S7) which in turn increased NEC (*P* < 0.0001; Fig. 5c, Table S8), creating a biophysical feedback loop. NCP and NEC were also positively correlated (*P* < 0.0001; Fig. 5b and Table S9), and there was a significant interaction with site for all three models (*P* < 0.002; Fig. 5, Tables S7–S9). Interestingly, the effect of NCP on NEC was strongest at Bob Creek, the site with the highest abundance of macrophytes and also highest calcification rates (Table S10). Furthermore, dissolved oxygen (DO) and pH were highly correlated (*P* < 0.001, R^2^ = 0.72; Figs S1, S2), indicating that changes in pH are tightly coupled with production and respiration rates.

## Discussion

The coastal systems studied here exhibited extreme spatiotemporal variability in pH that is both affecting and largely driven by biological processes. Specifically, producer dominance and ecosystem metabolism had a commanding influence over the mean and range in tide pool pH (Figs 3 and 4a,c), and this pattern persisted across unique biological and physicochemical environments at sites spanning ~1,800 km of coastline (Fig. 2, Table S2). Pools with the highest relative amount of non-encrusting producers always had the highest and most variable pH environments, and had significantly different mean pH values between daytime and nighttime sampling events (Figs 3, 4). This relationship holds true across all 57 tide pools, independent of latitude. Two processes likely drove the major differences in daily pH variability between producer- and consumer-dominated pools. First, both producers and consumers always respire, whereas producers also photosynthesize, increasing pH during daylight hours. Therefore, nighttime respiration rates were likely driven by the total biomass of organisms in each pool as opposed to daytime when pH conditions diverged between producer- and consumer-dominated pools (Fig. 4c). Second, nighttime respiration was limited by oxygen availability as pools became rapidly hypoxic at night (Fig. S2). pH quickly stabilized (i.e., the difference in pH between sampling times decreased) and average pH became similar across pools regardless of producer dominance or tide pool physical attributes (Figs 1 and 4c,d). These results in a dynamic natural ecosystem are consistent with findings from controlled laboratory experiments that fleshy macroalgae can increase mean and variance in pH^35,36^, highlighting the exceptional ability of producers to control local pH conditions.

The relationships between pH, NCP, and NEC illustrate the importance of biophysical feedbacks in coastal ecosystems (Fig. 5). Here, pH both drove and mediated critical ecosystem functions. The fact that producer dominance was a significant driver of both pH and ecosystem metabolism further highlights the key role of community composition in determining the strength of this biological feedback loop. However, other environmental conditions also vary across coastal systems and can influence the feedbacks between pH and ecosystem processes. For example, mean pH was related to pool size (Fig. 3, Table S4), a factor controlling seawater residence time in the pools which is known to amplify or dampen biologically-driven pH variation^37^. Increased temperature also tended to increase pH mean (Fig. 3), possibly by decreasing CO_2_ solubility. Environmental conditions can also indirectly influence pH dynamics as multiple parameters including temperature, light availability, and nutrients mediate NCP and NEC rates^17,38,39^. Such indirect effects are missing from many current OA projections that are based on open ocean data and from laboratory experiments that attempt to minimize factors driving pH variability (but see^40–42^). Several of these parameters are predicted to change in the future along with ocean pH, underscoring the need to incorporate natural variability and feedbacks from multiple parameters in OA projections.

The California Current System has extensive spatiotemporal variability in its physicochemical environment, especially with respect to pH^43,44^, likely due to regional differences in upwelling dynamics^45^. From our southern to northern study sites, which spanned ~11° in latitude, mean oceanic pH decreased from 8.12 to 7.87 (Table S2). However, tide pool pH was de-coupled from ocean pH during low tide, highlighting the capacity for local communities to buffer changes in open ocean chemistry. For example, communities at the northernmost site (Bob Creek) experienced the lowest ocean pH, but also had the highest tide pool pH (Fig. 1a,b) and highest within-site pH variability, including a 3 orders of magnitude increase in pH range from the most stable pool (7.16–7.29) to the most variable pool (7.14–8.9). Notably, a change in pH of 1.77 units over 12 hours in the most variable pool is nearly 9 times the predicted change in pH over a century^8^. This is because, while having the highest cover of calcifying invertebrates (mostly mussels *Mytilus californianus*; Fig. 2b) and highest average NEC rate (Table S10), Bob Creek pools also contained the greatest cover of non-encrusting producers (Fig. 2b). Specifically, the pool that had the highest relative amount of non-encrusting producers was 0.85 pH units higher than the adjacent ocean (Fig. 4e). Notably, the pool with the highest relative cover of consumers (also at Bob Creek) also deviated substantially from the ocean, dropping pH by 0.4 units relative to the ocean (Fig. 4e). Therefore, the community composition of the pools is driving pH further away from background conditions. Indeed, further research on how these communities respond to changing global conditions over the next 50–100 years—and especially how time averaged pH of each tide pool will track ocean pH conditions—is necessary for understanding how local pH conditions will change in the future.

Conversely, Corona del Mar, a site dominated by encrusting corallines with little fleshy macroalgal or invertebrate cover (Fig. 2b), had the lowest variability in tide pool pH with less than an order of magnitude increase in the pH range from the most stable pool (7.76–8.26) to the most variable pool (7.58–8.56) (Fig. 1g,h). pH values were most similar between tide pools and the adjacent ocean at Corona del Mar (difference between tide pool and ocean pH ranged from −0.02 to 0.31 across all pools), suggesting that these systems are more likely to be affected by changes in average ocean pH than pools at the other three sites. As producer dominance is a crucial driver of this decoupling of local pH from oceanic conditions, alterations to community composition via human disturbance^46,47^ or global changes in temperature^47,48^ are likely to diminish the capacity of coastal ecosystems to locally buffer OA and maintain ecosystem functioning.

Prior studies have suggested that producer-dominated systems, such as seagrass beds and kelp forests, could act as local refugia from OA because of their ability to elevate daytime pH^49,50^. However, it is still unclear whether increasing daytime pH (and diel variability) can benefit calcifiers when high nighttime respiration rates in these systems create corrosive conditions. A recent laboratory study showed higher calcification rates in oscillating relative to stable pCO_2_ environments in corals, possibly because they can store carbon (in the form of HCO_3_^−^) at night when pCO_2_ is high^51^. Another field study demonstrated that calcification may be more related to the number of hours spent at high pH rather than the mean^52^. Further, a recent study demonstrated that the mussel, *Mytilus edulis*, can increase calcification rates in the presence of dense macrophyte communities by shifting calcification activity to the day when pH is high^53^. Here, calcifiers experienced extreme swings in pH over a diel cycle (Fig. 1) and were consistently exposed to under-saturated waters at night (Table S2), yet the tide pool systems still maintained high abundances of invertebrate calcifiers and high rates of daytime NEC (Table S10). In other words, invertebrate calcifiers are not just persisting in these extremely variable pH conditions; they are thriving, suggesting that pH buffering is important even under present day conditions. It is important to note, however, that local pH buffering will not adequately alter global changes in ocean pH^54^. Further, long-term studies are needed to quantify the response of tide pool pH to global changes in ocean acidity.

Interestingly, we documented a latitudinal pattern in community composition that parallels the current understanding of how different types of algae respond to ocean pH. Specifically, ocean pH decreased from south to north, and across this gradient, there was a transition from high crustose coralline cover to high fleshy macroalgal cover (Fig. 2b). Coralline algae are highly sensitive to pH^55^, and studies at natural CO_2_ vents have shown a shift from coralline to fleshy algal dominance in closer proximity to these vents^56,57^. The gradient in ocean pH from south to north could be facilitating the shift from calcifying to non-calcifying algae, which in turn could be creating a favorable environment for mussels. (Notably, nutrients and several other factors also varied across our latitudinal gradient and nutrient availability can differentially affect fleshy and coralline algae^58–60^). Mussels are also the dominant calcifiers in other low and variable pH environments, such as in the Kiel Fjord^61^ and, therefore, may be better at withstanding “harsh” pH environments than other calcifiers. However, pH conditions in this study were only measured during low tide, when the tide pools are separated from the ocean. During high tide, the mussels (and other calcifiers) may get a reprieve from corrosive pH environments, which could also be facilitating their persistence.

This study explicitly included the natural variability of coastal ecosystems, rather than attempting to control it, finding strong and consistent evidence for biological drivers of pH conditions. Previous laboratory studies, primarily focused on coral reef organisms, demonstrated that individuals^36^ and community interactions^35,42^ can alter local pH, and targeted field studies have quantified the contribution of either ecosystem metabolism or community composition to local pH in coral reef^22,24,26,27,62^, seagrass bed^49,50^, kelp forest^50,63^, and rocky intertidal systems^17,29,63^. The present study, however, reveals the importance of community composition in mediating biological feedbacks at sites across a latitudinal gradient with unique physical, biological, and chemical environments. Further, we show here that biological feedbacks have a major impact on ecosystem functioning, as pH is both driven by and mediating ecosystem metabolism. The robustness of this feedback loop is a conduit for ecosystems to naturally buffer themselves from the increasing threat of ocean acidification.

## Materials and Methods

### Study Sites and Tide Pool Characterization

To test the relative effect of producer dominance, ecosystem metabolism and physical attributes on pH variability, tide pool habitats were characterized at four sites from southern California to central Oregon spanning approx. 11 degrees of latitude: Corona del Mar, CA (n = 14 pools), Monterey Bay, CA (n = 13 pools), Bodega Bay, CA (n = 15 pools), and Bob Creek, OR (n = 15 pools, Fig. 1). All data were collected between July and September 2016. Pools were selected haphazardly within the mid to high intertidal zone, assuring a range of different physical and biological characteristics to represent the spectrum of local habitats.

Tide pools were characterized in terms of both their physical characteristics and their biological communities. The measured physical attributes of each pool included tide height, maximum depth, water volume, perimeter, bottom surface area, and ratio of bottom surface area to volume. Tide heights were surveyed with a laser level and were normalized to the maximum tidal extent at each site to account for variation in tidal amplitude across the latitudinal gradient. Maximum depth was measured with a standard ruler at the deepest point in the pool, and pool volume was estimated using dye dilution assays on a spectrophotometer^64^. A flexible mesh quadrat with 10 × 10 cm squares was placed over the bottom of each pool to estimate surface area^65^, and a demarcated chain was spread along the edge of each pool to measure perimeter.

To determine community composition, water was temporarily removed from each pool with a hand-pump and a flexible mesh quadrat was spread along the bottom. Percent cover of all sessile organisms was estimated by visual surveys^65^, while individual mobile organisms were counted and converted to percent cover as described in the supplemental text (Supplemental Methods). Taxa were identified to the lowest possible taxonomic unit (Table S1) and then grouped as either producers or consumers for calculation of “producer dominance”. Community percent cover could exceed 100% due to canopy layering within the pools, and relative cover of each functional group was calculated as the percent cover of each group divided by total cover in each pool. All physical and biological measurements were taken at least 24 h before the water sampling events, ensuring that the tide pools were flushed with ocean water at least twice before water sampling occurred.

### Biogeochemical Characterization of Tide Pool Seawater

To characterize the physicochemical environments within pools and calculate metabolic rates, daytime and nighttime water sampling was conducted during low tide at each site. All data from this study were collected in a hierarchical design with time points (five or six samples) nested within time of day (day or night), tide pools, and sites. In each pool and the adjacent ocean, discrete water samples (400 ml) were collected hourly through tubing connected to a sealed plastic Erlenmeyer flask using a hand-pump over a 6-hour period (note: samples were collected only during a 5-hour period at night in Corona del Mar due to the timing at which pools were flushed by the rising tide). Tide pool water samples were collected right above the benthos, and adjacent ocean samples were collected at the surface. Each water sample took approx. 5 minutes to collect and process. Simultaneously, temperature, dissolved oxygen (DO), and salinity were measured using a multi-parameter and ODO pro meter (YSI, Yellowsprings, OH, USA). YSI probes were calibrated within 24 h of each sampling period using standard calibration solutions. Wind speed was measured hourly using a hand-held anemometer (HoldPeak HP-846A, Hong Kong). Continuous measurements of temperature and light intensity were recorded at 1 min intervals using HOBO TidbiT temperature loggers and Pendant light loggers bolted to the center of each tide pool (Onset Computer Corp., Bourne, MA). Light intensity (lumens m^−2^) was converted to photosynthetically active radiation (PAR; µmol photons m^−2^ s^−1^) following methods by Long *et al*.^66^.

Each discrete water sample was immediately divided into separate storage containers for analysis of pH, total alkalinity (TA), and dissolved inorganic nutrients (NH_4_^+^, NO_3_^−^, PO_4_^3−^). All sampling and storage containers were cleaned with 10% HCl, rinsed with MilliQ water, and rinsed three times with sample water. pH (total scale) was measured immediately in 50 ml of seawater using an Orion Star Multiparameter Meter with a ROSS Ultra glass electrode (Thermo Scientific, USA, accuracy = ±0.2 mV, resolution =  ±0.1, drift < 0.005 pH units per day) and a traceable digital thermometer (5-077-8, accuracy = 0.05 °C, resolution = 0.001 °C; Control Company, Friendswood, TX, USA) for multipoint calibration to a tris standard from the Dickson Lab at Scripps Institution of Oceanography following Dickson SOP 6a^67^. The glass electrode used to measure mV (converted to pH) was calibrated within 24 h of each sampling event. TA samples were stored in 250 ml brown Nalgene^®^ bottles and fixed with 100 µl of 50% saturated HgCl_2._ The remaining water was filtered through GF/F filters (0.7 µm) with a syringe, stored in 50 ml centrifuge tubes, and frozen at −20 °C for dissolved inorganic nutrient analyses. *In situ* pH and the remaining carbonate parameters were calculated using the *seacarb* package in R^68^. We note that error propagation for calculating Ω_arag_ based on pH and TA is ~3.6% and longer-term studies should consider using TA and DIC^69^.

### Sample Processing

Total alkalinity samples were analyzed using open-cell potentiometric titrations on a Mettler-Toledo T50 auto-titrator following Dickson SOP 3b^67^. A certified reference material (CRM) from the Dickson Lab at the Scripps Institution of Oceanography was analyzed at the beginning of each sample set. The accuracy of the CRMs never deviated more than ± 0.8% from the standard value, and TA measurements were corrected for these deviations. NO_3_^−^ and PO_4_^3−^ analyses were conducted on a QuickChem 8500 (Lachat, USA), and NH_4_^+^ was analyzed using the phenol-hypochlorite method on a UV-1800 Shimadzu spectrophotometer^70^, with standard curve R^2^ values of >0.99 in all cases.

### Net Ecosystem Calcification and Net Community Production Calculations

The total alkalinity anomaly technique^71^ was used to calculate hourly measurements of net ecosystem calcification (mmol CaCO_3_ m^−2^ hr^−1^) with the following equation:

1 $$NEC=\frac{\Delta TA\,\cdot \rho \cdot V}{2\cdot SA\cdot t}$$

ΔTA/2 is the difference in TA (mmol kg^−1^) between two consecutive time points, with one mole of CaCO_3_ formed per two moles of TA; *ρ* is the density of seawater (1023 kg m^−3^); *V* is the volume of water in the pool at each time point (m^3^); *SA* is the bottom surface area of the tide pool (m^2^); and *t* is the time between sampling points (h). TA was normalized to a constant salinity of 36 units to account for changes in evaporation, and then corrected for dissolved inorganic nitrogen and phosphorus to account for their small contributions to the acid-base system^72^. Positive NEC values represent net calcification while negative values represent net dissolution.

Differences in dissolved inorganic carbon (DIC), calculated from pH and TA using the *seacarb* package in R^68^, were used to calculate net community production rates (mmol C m^−2^ hr^−1^) using the following equation^73^:

2 $$NCP=\frac{{\rm{\Delta }}DIC\cdot \rho \cdot V}{SA\cdot t}-NEC-FC{O}_{2}$$

ΔDIC is the difference in salinity-normalized DIC (mmol kg^−1^) between consecutive time points, and NEC is subtracted to account for changes in DIC by the precipitation or dissolution of CaCO_3_. Positive values represented net photosynthesis while negative values indicate net respiration during that time interval. *FCO*_2_ (mmol m^−2^ hr^−1^) is subtracted to account for the air-sea flux of CO_2_ and is calculated as:

3 $$FC{O}_{2}=k\cdot s\cdot \rho (C{O}_{2-water}-C{O}_{2-air})$$

where *k* is the gas transfer velocity (m h^−1^), and *s* is the solubility of CO_2_ in seawater calculated from *in situ* temperature and salinity^74^. CO_2_ in air was assumed to be 400 µatm based on concurrent measurements at the Mauna Loa Observatory^75^. The CO_2_ transfer velocity was based on wind measurements from the handheld anemometer using the parameterization by Ho *et al*.^76^.

### Data Analysis

Linear mixed effects models were used to (i) test the relative contribution of biological and physical processes to pH variability, (ii) determine the relationship between producer dominance and the de-coupling of ocean and tide pool pH, (iii) determine the relationship between producer dominance and ecosystem metabolism, and (iv) assess the relationship between pH, NCP, and NEC in the tide pool ecosystems. To determine the relative contribution of producer dominance, ecosystem metabolism, tide pool physical attributes, temperature, and integrated PAR on pH variability in tide pools, we constructed two models. First, we modeled mean tide pool pH over the 5 or 6-h sampling period as the dependent variable with an interaction term for whether samples were collected during the day or night, and the second model evaluated range in pH over the entire sampling period (both day and night) as the dependent variable. To determine if producer dominance led to the de-coupling of local pH from ocean pH during low tide, we tested the relationship between producer dominance and the maximum difference between tide pool and ocean pH over the entire sampling period. We also evaluated the relationship between producer dominance and ecosystem metabolism. Site was included as a random intercept in all above models to account for site-level variability, and day/night was included as a random intercept in the mean model to account for repeated measures within each pool. To test the relationship between pH, NCP, and NEC across all time points, individual linear models were used with pool as a random effect and site as an interaction term. Lastly, variance in community composition both within and across sites was visualized with a nonmetric multidimensional scaling (NMDS) analysis using the *Vegan* package in R^77^.

We evaluated producer dominance and ecosystem metabolism as biological processes that could influence carbonate dynamics in the pools. Producer dominance was calculated as the relative percent cover of non-encrusting producers (macroalgae + surfgrass) minus percent cover of consumers (all invertebrates). Positive values indicate non-encrusting producer-dominated communities, whereas negative values indicate consumer-dominated communities. Encrusting algae were not included in this analysis because they had a very low contribution to producer biomass in the pools^78^ even though there was a high percent cover of them at the lower latitude sites. As net production rates are likely more dependent on biomass than percent cover, including them would have skewed the analysis. As a composite measure of ecosystem metabolism for each pool, we quantified the relationship between TA and DIC across all time points (Fig. S3), where TA represents relative changes in calcification versus dissolution (NEC) and DIC represents changes in production (NCP). TA/DIC slopes are commonly used as an integrative measure of NEC and NCP^20,62,79^, with higher TA/DIC slopes signifying greater changes in calcification/dissolution per unit production/respiration across the entire time-series within each tide pool (Fig. S3).

Six physical attributes of the tide pools, light availability, and temperature were assessed as physical parameters that could affect carbonate dynamics in the tide pools. To reduce dimensionality in the six physical attributes, we conducted a principal components analysis (PCA). The first two axes were used to describe differences in physical attributes in all subsequent analyses. PAR was integrated between the first and last water sample collected for each pool and represents the cumulative amount of light available in each pool. PAR was log transformed to meet model assumptions. Temperature was the mean or range over each sampling period for the mean and range pH models, respectively.

Normality and homoscedasticity were assessed visually for all models using quantile-quantile plots and box plots of residuals by grouping factors, respectively. Heteroskedasticity of the pH mean model (between day and night sampling events) led us to use the *VarIdent* function to allow for unequal variances between daytime and nighttime sampling events. Subsequent models met all model assumptions. We checked for multicollinearity using variance inflation factors, which were ≤2 for all predictors indicating no collinearity (Table S11). We tested for non-linearity in our models, but found none. Therefore, we used linear relationships in all statistical models. All mixed effects models were run using the *nlme* package in R^80^.

### Data accessibility statement

All data and code used for this study are available at https://github.com/njsilbiger/Biophysical_feedbacks_in_coastal_ecosystems.

## Electronic supplementary material

Supplemental Info
