# Supplementary material for: Biophysical feedbacks mediate carbonate chemistry in coastal ecosystems across spatiotemporal gradients
Source: Sci Rep. 2018 Jan 15;8:796. doi: 10.1038/s41598-017-18736-6 (PMC5768679; doi:10.1038/s41598-017-18736-6)
Supplement: Supplementary file 1 — Supplemental Info [file 41598_2017_18736_MOESM1_ESM.pdf]

Title: Biophysical feedbacks mediate carbonate chemistry in coastal ecosystems across spatiotemporal gradients

Authors: Nyssa J. Silbiger and Cascade J. B. Sorte

Supplement

Methods for estimating surface area of mobile organisms from individual counts
Tables S1 – S11
Figures S1 – S3

Methods for estimating surface area of mobile organisms from individual counts
Individual count data for all mobile organisms were converted to percent cover by estimating 2-dimensional surface area. Turban snails (n = 56), periwinkle snails (n = 56), limpets (n = 82), and chitons (n = 46) were collected from tide pools in Corona del Mar, CA between January and March, 2017. All individuals were photographed next to a standard ruler, and the surface area was calculated using ImageJ software. We then averaged the individual surface areas for each group (turban snails = 2.56 cm^2 ± 0.06 SE, periwinkle snails = 0.28 cm^2 ± 0.008 SE, limpets = 1.85 cm^2 ± 0.13 SE, and chitons = 1.20 cm^2 ± 0.06 SE) and multiplied these mean values by the number of individuals in each tide pool across sites. These four groups made up 98% of all mobile organisms in our dataset. The remaining organisms were classified as follows: whelks and grapsid crabs = 2 cm^2; urchins, brittle stars, ochre sea stars, and cancer crabs = 10 cm^2. Overall, the total percent cover of mobile organisms in each pool ranged from 0.21 – 9.45 % (mean: 2.7 % ± 0.28 SE) and, thus, mobile organisms were minor contributors to total live cover in the tide pools.

Supplemental Figures

Figure S1: pH and dissolved oxygen in tide pools at sites on the U.S. west coast. Circles represent pH and DO from all time points across all sites (n = 768). pH and dissolved oxygen (mg L^{-1}) are highly co-linear ($F_{1,726}$ = 1843, p<0.001, R^2 = 0.72). Data are colored by site (see Fig. 1): Corona del Mar, CA is lightblue, Monterey Bay, CA is royal blue, Bodega Bay, CA is magenta, and Bob Creek, OR is red.

Figure S2: Dissolved oxygen time-series. DO (mg L^{-1}) versus time during the (a,c,e,g) daytime and (b,d,f,h) nighttime sampling for (a,b) Bob Creek, OR, (c,d) Bodega Bay, CA, (e,f) Monterey Bay, CA, and (g,h) Corona del Mar, CA. Data from tide pools are in black and the adjacent ocean samples are in grey.

Figure 3: Property-property plots of total alkalinity (TA) and dissolved inorganic carbon (DIC). Data are salinity-normalized TA versus DIC values, and the background is colored by aragonite saturation state: blue colors are low saturation states and pink are higher saturation states. Values < 1 are under-saturated with respect to aragonite. Changes in DIC over time represent photosynthesis and respiration and changes in TA represent changes in calcification and dissolution, where 2 mols of TA are taken up for every mol of $CaCO_3$ produced (see diagram in panel a). Each black dot is from a single time point. Lines are the best fit lines from a simple linear regression that included all points within a single tide pool (n = 12). The slopes of these lines represent the relative balance of net ecosystem calcification (NEC) to net community production (NCP): steeper slopes have a higher relative amount of NEC per unit NCP. Flat slopes represent minimal changes in NEC across the time-series. Panels are by site from north to south: (a) Bob Creek, OR, (b) Bodega Bay, CA, (c), Monterey Bay, CA, and (d), Corona del Mar, CA.

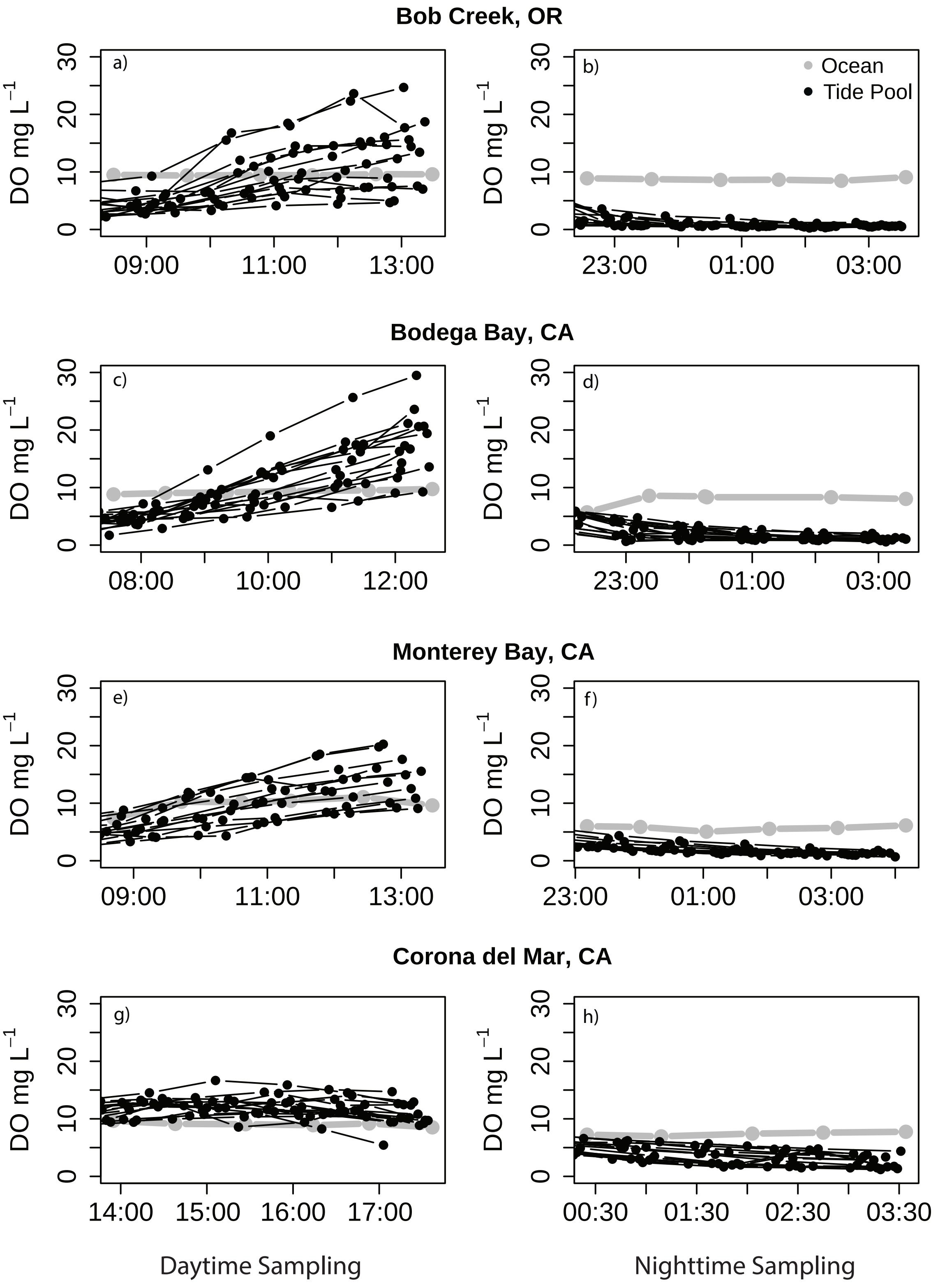
Bob Creek, OR
Bodega Bay, CA
Monterey Bay, CA
Corona del Mar, CA
a)
b)
c)
d)
e)
f)
g)
h)
Ocean
Tide Pool
DO mg L^{-1}
09:00
11:00
13:00
23:00
01:00
03:00
08:00
10:00
12:00
14:00
15:00
16:00
17:00
00:30
01:30
02:30
03:30
Daytime Sampling
Nighttime Sampling

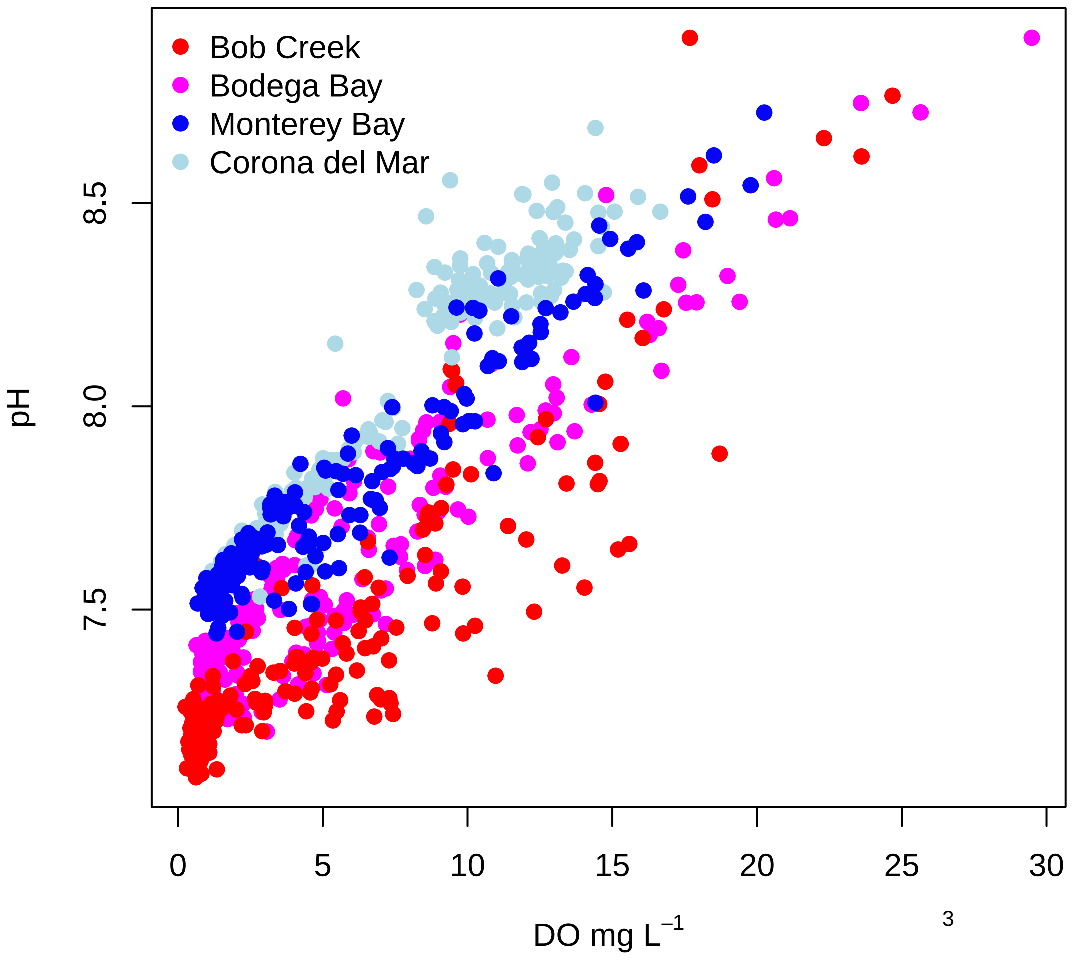

Bob Creek
Bodega Bay
Monterey Bay
Corona del Mar
pH
7.5
8.0
8.5
0
5
10
15
20
25
30
DO mg L–1
3

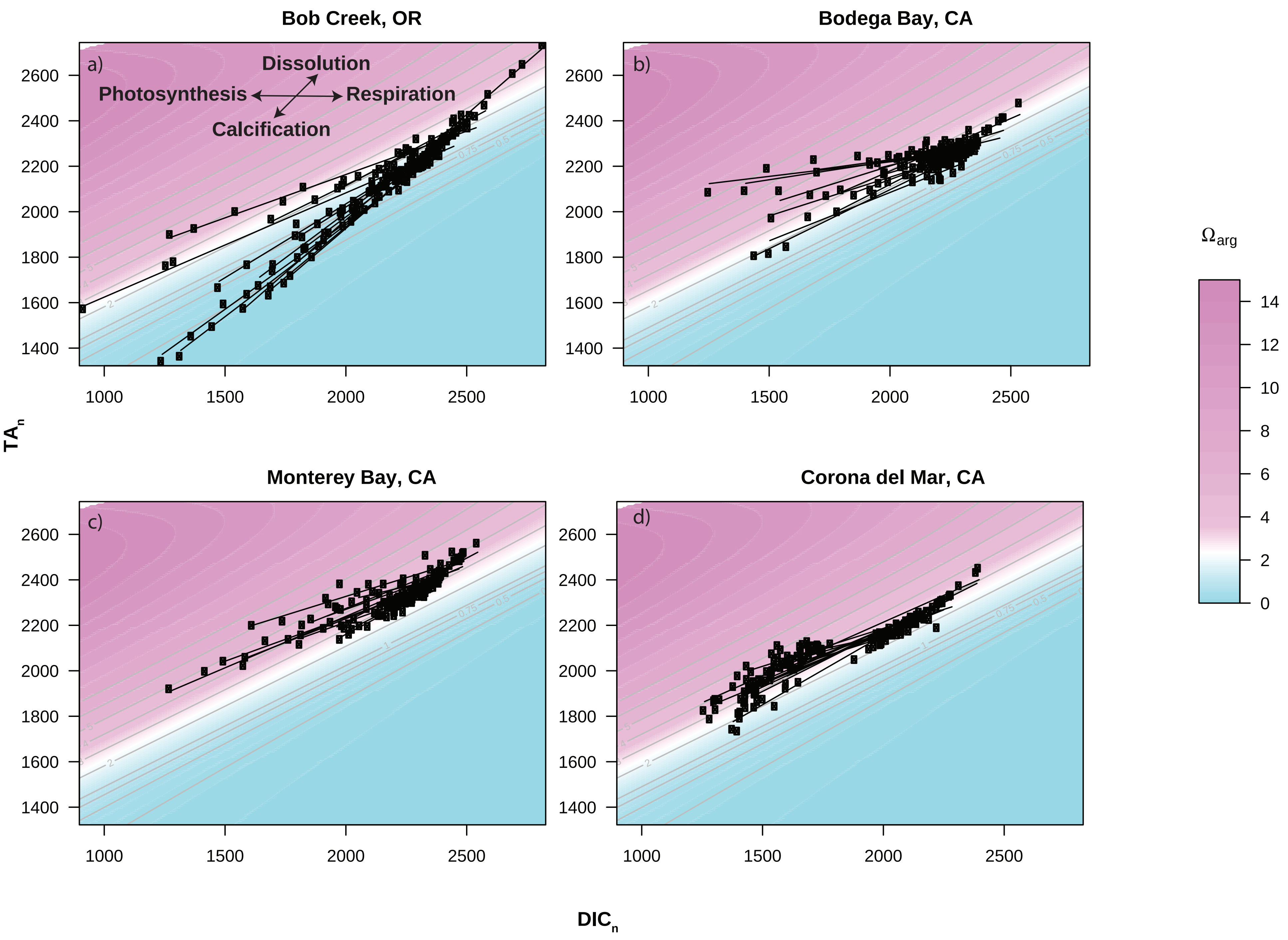
Bob Creek, OR
Bodega Bay, CA
Monterey Bay, CA
Corona del Mar, CA
a)
b)
c)
d)
Dissolution
Photosynthesis
Respiration
Calcification
TA_n
DIC_n
Ω_{arg}
2600
2400
2200
2000
1800
1600
1400
1000
1500
2000
2500
14
12
10
8
6
4
2
0

Table S1: Taxon for each site by (a) algae and grasses and (b) invertebrates.

Corona del Mar, California	Monterey Bay, California	Bodega Bay, California	Bob Creek, Oregon
**Algae and Grasses**			
Caulacanthis okamurae	*Chonracanthus exasperata*	*Cladophora spp.*	*Cladophora spp.*
Ceramium spp.	*Bossiella spp.*	*Bossiella spp.*	*Corallina spp.*
Chaetomorpha spp.	*Caulacanthis okamurae*	*Caulacanthis okamurae*	Crustose coralline
Colpomenia sinuosa	*Corallina spp.*	*Corallina spp.*	*Farlowia mollis*
Corallina spp.	Crustose coralline	Crustose coralline	*Neogastroclonicum subarticulatum*
Crustose coralline	*Cryptopluera violacea*	*Cryptopluera violacea*	*Mazzaella splendens*
Cystosyra spp.	*Neogastroclonicum subarticulatum*	*Endocladia muricata*	Non Coralline Crust
Dictyota flabellata	*Mastocarpus spp.*	*Fucus spp.*	*Polysiphonia spp.*
Ectocarpus spp.	*Mazzaella affinis*	*Neogastroclonicum subarticulatum*	*Prionitis spp.*
Egregia menziesii	*Mazzaella leptorhynchos*	*Mastocarpus spp.*	
Endocladia muricata	*Mazzaella parksii*	*Mazzaella flaccida*	
Neogastroclonicum subarticulatum	Non Coralline Crust	*Mazzaella splendens*	
Gelidium spp.	*Prionitis spp.*	Non Coralline Crust	
Laurencia pacifica	*Silvetia compress*	*Phyllospadix spp.*	
Non Coralline Crust	*Ulva californica*	*Prionitis spp.*	
Petalonia binghaminae			
Polysiphonia spp.			
Sargassum muticum			
Ulva californica			
**Invertebrates**			
Anthopleura spp.	*Anthopleura spp.*	*Anthopleura spp.*	*Anthopleura spp.*
Balanus/Chthamalus	*Balanus/Chthamalus*	*Balanus/Chthamalus*	*Balanus/Chthamalus*
Ceratostoma spp.	Brittle star	Turban snail *(Chlorostoma spp.)*	Turban snail *(Chlorostoma spp.)*
Turban snail *(Chlorostoma spp. or Agathistoma spp.)*	Cancer crab	*Littorina spp.*	*Cyanoplax spp.*
Cyanoplax spp.	Turban snail *(Chlorostoma spp.)*	*Lottia spp.*	*Littorina spp.*
Fissurella spp.	*Cyanoplax spp.*	*Mopalia spp.*	*Lottia spp.*
Littorina spp.	*Fissurella spp.*	*Mytilus spp.*	*Mopalia spp.*
Lottia spp.	*Littorina spp.*	*Nucella spp.*	*Mytilus spp.*
Mytilus spp.	*Lottia spp.*	*Pachygrapsus spp.*	*Nucella spp.*
Nuttalina spp.	*Mopalia spp.*	*Pagurus spp.*	*Pachygrapsus spp.*
Pachygrapsus spp.	*Mytilus spp.*	*Pisaster spp,*	*Pagurus spp.*
Pagurus spp.	*Nucella spp.*	Sponge	*Pisaster spp.*
Serpulorbis spp.	*Pachygrapsus spp.*	*Tetraclita*	*Pollicipes polymerus*
Tetraclita spp.	*Pagurus spp.*	Urchin	*Semibalanus spp.*
	Pisaster spp.		*Serpulorbis spp.*
	Urchin		Urchin

Table S2: Tide pool and site characteristics. Ranges in physical attributes and chemical parameters measured from the tide pools and adjacent open sample during the day and night. The ranges are data collected across all tide pools and time points within each site.

	Corona del Mar, CA	Monterey Bay, CA	Bodega Bay, CA	Bob Creek, OR
**Latitude**	33.587753	36.620795	38.316695	44.24257
**Longitude**	-117.867242	-121.942202	-123.071102	-124.11255
**Daytime Sampling Date**	9/16/2016	7/11/2016	7/25/2016	8/8/2016
**Nighttime Sampling Date**	9/27/2016	7/12/2016	7/27/2016	8/10/2016
**Ocean Chemistry (Day)**				
**Temperature (°C)**	19.2-19.7	12.2-13.5	10.6-12.7	9.3-11.2
**DO (mg L^{-1})**	8.52-9.65	7.4-11.06	8.82-9.74	9.38-9.61
**pH_T**	8.23-8.29	8.0-8.3	7.80-8.23	7.85-8.09
**TA (µmol kg^{-1})**	2118-2179	2170-2300	2173-2282	2190-2273
**DIC (µmol kg^{-1})**	1743-1821	1855-2089	1879-2163	1984-2151
**pCO_2 (µatm)**	190-221	182-436	225-722	331-641
**Ω_{arag}**	3.74-4.10	1.98-3.72	1.24-2.95	1.30-2.19
**NH_4^+ (µmol L^{-1})**	0.04-1.18	0.38-0.77	0.28-1.49	1.27-15.7
**NO_3^- + NO_2^- (µmol L^{-1})**	0-0.73	1.45-3.51	5.1-12.5	2.99-15.4
**PO_4^{3-} (µmol L^{-1})**	0.41-0.53	0.39-0.60	0.85-1.29	0.50-1.26
**Ocean Chemistry (Night)**				
**Temperature (°C)**	17.6-18.2	13.2-13.4	11.6-11.8	9.2-10
**DO (mg L^{-1})**	6.95-7.75	5.05-6.14	5.7-8.58	8.46-9.09
**pH_T**	7.91-8.01	7.79-7.93	7.87-8.02	7.70-7.74
**TA (µmol kg^{-1})**	2114-2190	2226-2279	2266-2305	2229-2459
**DIC (µmol kg^{-1})**	1918-1998	2082-2165	2075-2154	2158-2381
**pCO_2 (µatm)**	438-564	523-736	412-610	840-1011
**Ω_{arag}**	1.93-2.51	1.36-1.80	1.52-2.02	1.01-1.16
**NH_4^+ (µmol L^{-1})**	1.33-2.38	0.29-1.59	3.54-8.37	1.16-5.58
**NO_3^- + NO_2^- (µmol L^{-1})**	0.69-1.12	0.12-3.34	5.18-6.87	8.45-17
**PO_4^{3-} (µmol L^{-1})**	0.16-0.62	0.55-0.77	0.78-1.09	1.03-1.91
**Tide Pool Chemistry (Day)**				
**Temperature (°C)**	19.6-26.1	12.9-20	10.7-19.7	9.6-16.6
**DO (mg L^{-1})**	5.43-16.66	2.04-20.25	1.71-29.49	1.54-24.68
**pH_T**	8.12-8.68	7.45-8.72	7.20-8.90	7.20-8.91
**TA (µmol kg^{-1})**	1739-2129	1923-2524	1812-2357	1355-2432
**DIC (µmol kg^{-1})**	1260-1785	1272-2488	1251-2413	917-2515
**pCO_2 (µatm)**	65-274	38-1779	20-3092	15-3175
**Ω_{arag}**	3.00-5.89	0.67-5.91	0.34-7.65	0.31-5.62
**Tide Pool Chemistry (Night)**				
**Temperature (°C)**	16.7-25	12.9-14.6	11.9-13.2	10.3-14.9
**DO (mg L^{-1})**	1.19-7.17	0.68-5.46	0.57-7.26	0.26-6.74
**pH_T**	7.53-7.97	7.44-7.86	7.21-7.89	7.09-7.55
**TA (µmol kg^{-1})**	2052-2449	2253-2564	2135-2470	2008-2739
**DIC (µmol kg^{-1})**	1884-2396	2124-2545	2066-2537	2033-2816
**pCO_2 (µatm)**	490-1534	632-1832	565-2997	1335-4072
**Ω_{arag}**	0.93-2.23	0.68-1.59	0.35-1.56	0.26-0.85
**Tide Pool Physical Attributes**				
**Perimeter (m)**	1.95 - 7.15	1.82 - 5.79	1.45 - 6.41	1.98 - 5.81
**Max Depth (cm)**	5 - 44	12 - 45	7 - 40	15 - 32
**Surface Area (m)**	0.2 - 2.3	0.21 - 1.54	0.16 - 1.52	0.22 - 0.97
**Volume (L)**	3.73 - 127	9.85 - 96.75	7.62 - 97.88	4.81 - 8.15

Table S3: Summary results for model testing the effect of phyisical and biological drivers on pH range. Fixed effects are shown in panel (a) and random effects in panel (b). All variables were standarized. Bold values are statistically significant.

(a) Fixed Effects	Value	Std.Error	DF	t-value	p-value	
Intercept	-0.01	0.18	47	-0.06	0.95	
Producer Dominance (% Cover Producers - Consumers)	0.51	0.11	47	4.62	<**0.001**	
Temperature	0.12	0.19	47	0.07	0.94	
log(∫PAR)	0.11	0.13	47	0.93	0.36	
Pool Size (PC1)	0.09	0.06	47	1.35	0.18	
Pool Location (PC2)	0.02	0.10	47	0.24	0.80	
Ecosystem Metabolism (TA/DIC Slopes)	-0.35	0.16	47	-2.25	**0.03**	
(b) Random effects	StDev					
~1|Site						
Intercept	0.31					
Residual	0.74					

Table S4: Summary results for model testing the effect of phyisical and biological drivers on pH mean. Fixed effects are shown in panel (a) and random effects in panel (b). All variables were standarized. Bold values are statistically significant.

(a) Fixed Effects	Value	Std.Error	DF	t-value	p-value	
Intercept	0.68	0.19	98	3.51	**<.001**	
Producer Dominance (% Cover Producers - Consumers)	0.35	0.08	98	4.56	**<.0001**	
Day/Night	-1.30	0.26	3	-5.04	**0.02**	
Temperature	0.21	0.11	98	2.00	**0.05**	
log(∫PAR)	0.00	0.02	98	-0.26	0.79	
Pool Size (PC1)	-0.02	0.01	98	-2.65	**0.01**	
Pool Location (PC2)	0.02	0.01	98	1.44	0.15	
Ecosystem Metabolism (TA/DIC Slopes)	-0.27	0.12	98	-2.27	**0.03**	
Producer-Dominance x Day/Night	-0.37	0.08	98	-4.85	**<.0001**	
Ecosystem Metabolism x Day/Night	0.33	0.12	98	2.77	**0.01**	
(b) Random effects	StDev					
~1|Site						
Intercept	0.15					
~1|Day/Night x Site						
Intercept	0.31					
Residual	0.5327					

Table S5: Summary results for model testing the effect producer-dominance on pH divergence. Fixed effects are shown in panel (a) and random effects in panel (b). All variables were standarized. Bold values are statistically significant.

Fixed Effects	Value	Std.Error	DF	t-value	p-value	
Intercept	-0.08	0.05	52	-1.57	0.12	
Producer Dominance	0.008	0.001	52	5.27	**<0.001**	
Random effects	StDev					
~1|Site						
Intercept	0.078					
Residual	0.23					

Table S6: Summary results for model testing the effect producer-dominance on ecosystem metabolism. Fixed effects are shown in panel (a) and random effects in panel (b). All variables were standarized. Bold values are statistically significant.

Fixed Effects	Value	Std.Error	DF	t-value	p-value	
Intercept	0.54	0.09	52	6.06	**<0.001**	
Producer Dominance	-0.00186	0.00068	52	-2.72	**0.009**	
Random effects	StDev					
~1|Site						
Intercept	0.17					
Residual	0.11					

Table S7: ANOVA table for pH as a function of NCP x Site. Pool ID was included as a random effect

	numDF	denDF	F-value	p-value
Intercept	1	493	474510	**<.0001**
NCP	1	493	448	**<.0001**
Site	3	53	192.3	**<.0001**
NCP x Site	3	493	4.9	**0.002**

Table S8: ANOVA table for NEC as a function of pH x Site. Pool ID was included as a random effect

	numDF	denDF	F-value	p-value
Intercept	1	493	24.95	**<.0001**
pH	1	493	155.80	**<.0001**
Site	3	53	34.49	**<.0001**
pH x Site	3	493	5.98	**<.0001**

Table S9: ANOVA table for NEC as a function of NCP x Site.
Pool ID was included as a random effect

	numDF	denDF	F-value	p-value
Intercept	1	493	43.32	**<.0001**
NCP	1	493	555.74	**<.0001**
Site	3	53	8.90	**<.0001**
NCP x Site	3	493	22.25	**<.0001**

Table S10: Mean NCP and NEC values across all pools within each site during (a) day and (b) night sampling events.

Site	NCP (mmol m^{-2} hr^{-1})	NEC (mmol m^{-2} hr^{-1})
**(a) Day**		
**Corona del Mar, CA**	14.33	11.10
**Monterey Bay, CA**	34.42	6.92
**Bodega Bay, CA**	35.18	5.83
**Bob Creek, OR**	34.88	17.16
**(b) Night**		
**Corona del Mar, CA**	-18.28	-7.66
**Monterey Bay, CA**	-12.35	-4.95
**Bodega Bay, CA**	-13.57	-2.92
**Bob Creek, OR**	-9.16	-7.08

Table S11: Variance Inflation Factors used to test for co-linearity among predictor variables. All VIF values were ≤2.

**Parameter**	**VIF**
Producer Dominance	1.21
Temperature	1.89
PAR	1.27
Pool Size	1.16
Pool Location	1.15
Ecosystem Metabolism	1.48
